# Gutting the brain of inflammation: A key role of gut microbiome in human umbilical cord blood plasma therapy in Parkinson's disease model

**DOI:** 10.1111/jcmm.14429

**Published:** 2019-05-31

**Authors:** Jea‐Young Lee, Julian P. Tuazon, Jared Ehrhart, Paul R. Sanberg, Cesario V. Borlongan

**Affiliations:** ^1^ Center of Excellence for Aging and Brain Repair Morsani College of Medicine, University of South Florida Tampa Florida; ^2^ Department of Neurosurgery and Brain Repair Morsani College of Medicine, University of South Florida Tampa Florida; ^3^ Saneron CCEL Therapeutics, Inc. Tampa Florida; ^4^ Department of Pathology and Cell Biology Morsani College of Medicine, University of South Florida Tampa Florida; ^5^ Department of Psychiatry Morsani College of Medicine, University of South Florida Tampa Florida

**Keywords:** cord blood, gut microbiome, neurodegeneration, neuroinflammation, plasma

## Abstract

Current therapies for Parkinson's disease (PD), including L‐3,4‐dihydroxyphenylalanine (L‐DOPA), and clinical trials investigating dopaminergic cell transplants, have generated mixed results with the eventual induction of dyskinetic side effects. Although human umbilical cord blood (hUCB) stem/progenitor cells present with no or minimal capacity of differentiation into mature dopaminergic neurons, their transplantation significantly attenuates parkinsonian symptoms likely via bystander effects, specifically stem cell graft‐mediated secretion of growth factors, anti‐inflammatory cytokines, or synaptic function altogether promoting brain repair. Recognizing this non‐cell replacement mechanism, we examined here the effects of intravenously transplanted combination of hUCB‐derived plasma into the 1‐methyl‐4‐phenyl‐1,2,3,6‐tetrahydropyridine (MPTP)‐induced rat model of PD. Animals received repeated dosing of either hUCB‐derived plasma or vehicle at 3, 5 and 10 days after induction into MPTP lesion, then behaviourally and immunohistochemically evaluated over 56 days post‐lesion. Compared to vehicle treatment, transplantation with hUCB‐derived plasma significantly improved motor function, gut motility and dopaminergic neuronal survival in the substantia nigra pars compacta (SNpc), which coincided with reduced pro‐inflammatory cytokines in both the SNpc and the intestinal mucosa and dampened inflammation‐associated gut microbiota. These novel data directly implicate a key pathological crosstalk between gut and brain ushering a new avenue of therapeutically targeting the gut microbiome with hUCB‐derived stem cells and plasma for PD.

## 1 INTRODUCTION

Parkinson's disease (PD) ranks as the second‐most prevalent neurodegenerative disorder which affects the aging population.^1^ The major neuropathological hallmarks of PD consist of dopaminergic cell loss in the substantia nigra (SNpc) leading to a deficiency of dopamine in the striatum, and formation of intracellular inclusions, called Lewy bodies, which contain α‐synuclein aggregates.^2^ The clinical symptoms of PD have been routinely defined as progressive motor abnormalities, which include tremors, muscle rigidity, bradykinesia, impaired balance and freezing of gait.^1^ However, PD is also associated with many non‐motor symptoms, such as cognitive impairment, and abnormalities in the function of the gut, which contribute to overall morbidity of the disease.^1^, ^3^ Unfortunately, despite the scientific advances in our knowledge of disease pathology, there is no cure for PD, only relief from its symptoms. Current therapeutic applications to treat PD target pharmacological replacement of striatal dopamine (ie, Levodopa), coupled with non‐dopaminergic drugs to attenuate both motor and non‐motor symptoms. Additionally, deep brain stimulation has also been shown to provide therapeutic relief of symptoms.^1^, ^3^ Interestingly, an enriched environment has also exhibited potential for reducing oxidative damage and olfactory dysfunction in PD mice.^4^ Studies in stem cell‐based regenerative medicine, which also attempt to exogenously replace dopamine or stimulate endogenous host brain repair,^5^, ^6^ represents efforts towards disease‐modifying outcomes as well.

Cord blood plasma (CBP), which can be readily obtained from human umbilical cord blood (hUCB) during routine cell isolation, has mainly been considered a waste product. The potential trophic effects provided by CBP have been shown through culture supplementation of hUCB‐derived mesenchymal stem cells (MSCs),^7^ hUCB‐derived T‐lymphocytes,^8^ human dental cells^9^ or human endothelial colony‐forming cells^10^in vitro. Indeed, CBP may have underlying potential as a source of treatment for neurological diseases, given that hUCB cell grafts migrate to injury sites^11^ and emit growth factors that ameliorate ischaemia,^12^ hUCB mononuclear cells increase neurogenesis in aged rats,^13^ and neural cells derived from human placentas attenuate neuroinflammation and confer neuroprotection in PD rats.^14^ Additionally, in vivo studies determining the therapeutic potential of CBP in rats modelling acute ischemic stroke have been investigated with results showing enhancement of neurogenesis and reduced inflammation providing significant functional recovery post‐stroke.^15^ Moreover, α‐secretase extracted from CBP has also demonstrated the ability to improve cognitive deficits and amyloid plaque formation in Alzheimer's disease mouse models.^16^ Several studies have also investigated the composition of CBP with reports finding high amounts of various growth factors, along with a unique cytokine profile consisting of a low concentration of various pro‐inflammatory cytokines.^8^, ^17^, ^18^ Although CBP can have beneficial effects on cells in culture, the ability of CBP to elicit therapeutic benefits in models of neurodegenerative disorders must be determined before developing clinically relevant CBP‐based therapies.

Since inflammation has been connected to the progressive exacerbation of neurological diseases 19 and the inflammatory gut microbiome has recently been linked to a number of neurological disorders,^20^, ^21^, ^22^, ^23^, ^24^ including Parkinson's,^25^, ^26^, ^27^ this study was designed to analyse the gut for MPTP‐induced PD‐related dysfunction and to determine the therapeutic potential CBP at reducing the gut inflammation that may be present in this particular model. Findings from this study may aid in providing further support to the notion that a non‐dopaminergic and non‐CNS organ may contribute to PD pathology.^21^, ^23^, ^28^ Additionally it is of specific interest to determine if intravenous (iv) delivery of CBP could provide therapeutic benefit in both gut and brain tissues. This would provide valuable translational guidance for the development of a stand‐alone CBP therapeutic or quite possibly a combined CBP with stem cell therapy in targeting the gut microbiome for PD treatment.

The aim of this study was to determine if CBP alone is beneficial in reducing PD‐associated pathology in the brain and gut induced by intraperitoneal (ip) administration of an MPTP insult. Since the full efficacy of a CBP‐based therapy might not be ascertainable using a single injection, multiple administrations of CBP were provided over a 14 day period. To assess the efficacy of this treatment plan the motor, neurological and non‐motor related pathologies of PD assessed at baseline (day 0) and at days 1, 3, 7 and 14 after MPTP administration. Tissue pathology was examined using immunohistochemistry to help determine the extent of the MPTP‐induced nigral lesion and resultant inflammation in both the brain and intestinal tissues. Finally the populations of several inflammatory gut‐related microbiota were assayed to determine the relative inflammatory state of the gut microbiome. These study results provide insight into the potential of a CBP therapy for the treatment of neurodegenerative diseases.

## 2 MATERIALS AND METHODS

### 2.1 hUCB plasma

Frozen units of hUCB plasma (CBP) were provided by Saneron CCEL Therapeutics, Inc The CBP was aseptically isolated using the Sepax 2 fully automated cell processing system (Biosafe America Inc, Houston, TX), and tested for sterility using the BacT/ALERT microbial detection system (bioMérieux, Durham, NC) prior to freezing.

### 2.2 Animals

All experimental procedures were approved by the University of South Florida Institutional Animal Care and Use Committee (IACUC). Male Sprague‐Dawley rats (8 weeks old, n = 30) were housed under normal conditions (20°C, 50% relative humidity, and a 12‐hour light/dark cycle). All necessary precautions were taken to reduce pain and suffering of animals throughout the study. Animals were closely checked daily throughout the 2 week study period. All studies were performed by personnel blinded to the treatment condition.

### 2.3 Preparation of the animal model

**Figure 1:**
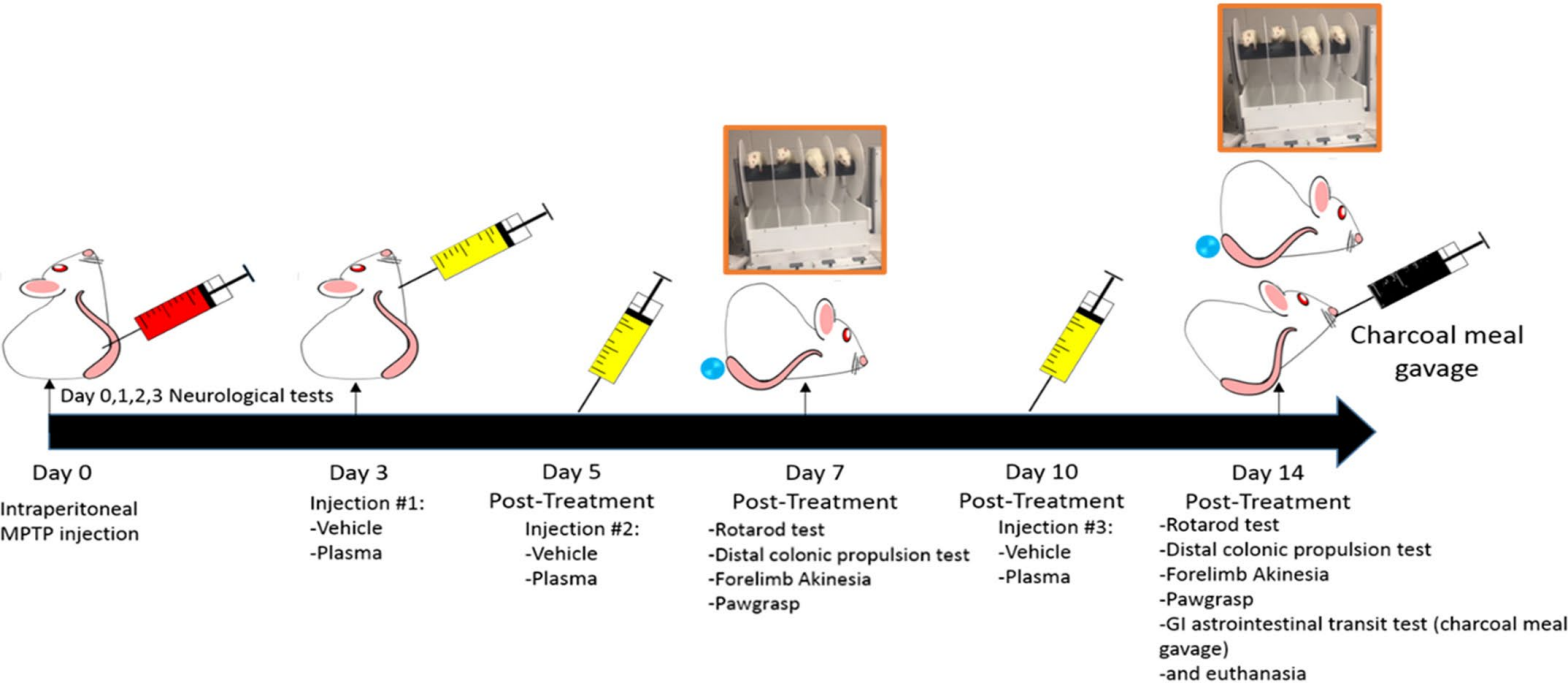
Schematic representation of experimental design. Animals received 1‐methyl‐4‐phenyl‐1,2,3,6‐tetrahydropyridine (MPTP) insult at day 0, and injections of cord blood plasma on days 3, 5 and 10 post insult. Animals underwent behavioural testing to document neurological and motor deficits at days 7 and 14 post MPTP induction of Parkinsonian pathology

To induce degeneration of nigrostriatal dopaminergic neurons in 8‐week‐old male Sprague‐Dawley rats, 100 µL of MPTP (20 mg/kg; Sigma Aldrich, St. Louis, MO) was intraperitoneally (ip) injected every 2 hours for a total of three injections per animal in both vehicle (n = 10) and CBP (n = 10) groups; sham animals (n = 10) received 0.9% saline (100 µL). All surgical procedures were performed under sterile conditions. On days 3, 5 and 10 post‐lesion, the plasma group received CBP (500 µL) iv with the sham and vehicle groups both receiving PBS iv (500 µL; Figure 1). Animal weight was recorded at the onset of the study, and monitored over the next three consecutive days post‐MPTP insult.

### 2.4 Behavioural testing

Behavioural tests for the observation of parkinsonian‐like motor and non‐motor deficits were administered at intervals prior to, 7 days post and 14 days post MPTP lesion. Standard behavioural testing included rotarod, forelimb akinesia and paw grasp were performed to observe generation of the classical motor‐function deficits and neurological dysfunction seen in PD. Distal colonic propulsion and gastric emptying studies (described below) were performed to observe changes in the gut associated non‐motor symptoms of PD.

### 2.5 Distal colonic propulsion

To measure colonic propulsion on day 7 and day 14 after MPTP administration, a single 5‐mm‐diameter glass bead was inserted into the distal colon to a distance of about 2 cm from the anus of each rat. After bead insertion, the rats were replaced individually without food and water in their home cages. Thereafter the animals underwent observation to ensure they showed normal behaviour, and the amount of time necessary for excretion of the glass bead was measured. The time required for expulsion of the glass bead, called mean expulsion time (MET), was determined (to the nearest 1.0 second) for each rat and the inhibition of colonic propulsion measured as the increase in MET versus control rats. The higher the MET value, the stronger the inhibition of colonic propulsion.

### 2.6 Gastric emptying

At the study termination day, all groups were provided an oral solution of charcoal (10%) in acacia gum (2%) by gavage. Animals were returned to their home cages for 30 minutes following oral administration, then euthanized by carbon dioxide inhalation. The intestines were then exteriorized and the distance traveled by the charcoal meal was measured, in addition to the full length of the intestines (from the pylorus through the anus). Results are expressed as distance traveled in centimeters as well as percent of distance traveled relative to the full length of the intestines.

### 2.7 Tissue collection

Under deep anesthesia, the rats were sacrificed at 14 days after MPTP insult. Animals were perfused transcardially with approximately 200 mL of ice cold PBS followed by 200 mL of 4% paraformaldehyde in PBS. The intestines and brains of these animals were removed and post‐fixed in the same fixative for 24 hours at 4°C. These tissues were then transferred to 30% sucrose in PB for at least 16 hours prior to tissue sectioning. Coronal sections were cut (40 µm) using a cryostat, collected and stored in a cryoprotectant solution at −20°C.

### 2.8 Immunohistochemistry

Staining tyrosine hydroxylase (TH) conducted on every sixth coronal section throughout the entire substantia nigra (SN). In all animals, sections were anatomically matched. Eight free‐floating coronal sections from each rat were washed three times in 0.1 M PBS to remove traces of the cryoprotectant. Afterwards, all sections were incubated in a 0.3% hydrogen peroxide (H_2_O_2_) solution for 20 minutes, then washed with PBS three times at 10 minutes each. Next, all sections were incubated for 1 hour in a blocking solution composed PBS supplemented with 3% normal goat serum and 0.2% Triton X‐100. Sections were then incubated overnight at 4°C with rabbit anti‐rat TH (1:100 TH, AB152; Millipore, Burlington, MA) antibody diluted in PBS supplemented with 3% normal goat serum and 0.1% triton X‐100. Sections were then washed three times with PBS and incubated for 1 hour in biotinylated goat anti‐rabbit secondary antibody (1:300; Vector Laboratories, Burlingame, CA) diluted in PBS supplemented with normal goat serum, and 0.1% Triton X‐100. Next, the sections were incubated for 60 minutes in avidin‐biotin substrate (ABC kit; Vector Laboratories) and washed with PBS three times at 10 minutes each wash. All sections were then incubated for 1 minute in 3, 30‐diaminobenzidine (DAB) solution (Vector Laboratories) and washed three times with PBS for 10 minutes each wash. Sections were then mounted onto glass slides, set to dry for 24 hours and dehydrated with increasing concentrations of ethanol (70%, 95% and 100%) for 2 minutes each and one time in xylenes for 2 minutes. The slides were then cover‐slipped using Permount mounting medium (Fisher Scientific, Pittsburg, PA). Control studies included exclusion of primary antibodies substituted with 3% normal goat serum in PBS. No immunoreactivity was observed in these controls.

### 2.9 Immunofluorescence

Immunofluorescent staining for the inflammatory markers of major histocompatibility complex (MHCII) and tumor necrosis factor alpha (TNF‐α) were conducted on every sixth coronal section throughout the entire SN. In all animals, sections were anatomically matched. Eight free‐floating coronal sections (40 mm) from each rat were washed three times in 0.1 M PBS to remove traces of the cryoprotectant. The sections were then incubated in blocking solution for 1 hour using PBS supplemented with 10% normal horse serum and 0.1% Triton X‐100. Sections were then incubated overnight at 4°C with mouse anti‐rat MHC II (anti‐RT1B (OX‐6); NB100‐65541; Novus Biologicals, Centennial, CO) or rabbit anti‐rat TNF‐α (ab6671; Abcam, Cambridge, MA) antibodies in PBS supplemented with 10% normal horse serum and 0.1% triton X‐100. Sections were washed three times for 10 minutes in PBS and then incubated in PBS supplemented with 10% normal horse goat serum and 0.1% Triton X‐100 containing corresponding secondary antibodies, goat anti‐rabbit IgG‐Alexa 488 (green; 1:500; Invitrogen) and goat anti‐ mouse IgG‐Alexa 594 (red; 1:750; Invitrogen), for 90 minutes. Finally, sections were washed five times for 10 minutes in PBS, and cover‐slipped with Fluoromount (Sigma; F4680). Sections were analyzed in independent channels with an Olympus FV1000 laser scanning confocal microscope equipped with Fluoview SV1000 imaging software. Control studies included exclusion of primary antibodies substituted with 3% normal horse serum in PBS. No immunoreactivity was observed in these controls.

### 2.10 Gut microbiome analysis

The presence and concentration of specific inflammatory, potentially harmful, microbiotic species residing within the gut was determined. Fluorescent in‐situ hybridization (FISH) analysis was used to identify the specific microbiota within the feces of the rats in this study. Fecal matter (1 g) collected during tissue processing was suspended in 2 mL of PBS and homogenized by repeated pipetting with a 1mL micropipettor and mixed using a vortex. Four per cent paraformaldehyde (6 mL) was added to the homogenized feces and stored overnight at 4°C. The tubes were then centrifuged at 10 *g* for 5 minutes to sediment undigested material. The bacterial laden supernatant was transferred a new tube and the centrifugation step was repeated. Following the final centrifugation the supernatant was transferred to a new tube and centrifuged at 60 *g* for 5 minutes to pellet any bacteria present. The supernatant was discarded and the pellet was resuspended in 500 µL of hybridization solution comprised of 50% (v/v) formamide, 100 µg/mL salmon sperm DNA, 5× saline‐sodium citrate buffer, and 0.1% (v/v) Tween 20. After a 30 minutes incubation at 37°C, 50 µL volumes of each sample were combined with 2.7 µL of fluorescently labelled oligonucleotides in a new tube and incubated in the dark for 2.5 hours at 45°C. The tubes were then centrifuged at 60 *g* for 5 minutes, the supernatant discarded, and the pellet was washed by resuspending in 20 µL of 0.1 SSC. This wash step was repeated an additional two times. After the final wash, the samples were resuspended in 20 µL of PBS, and centrifuged at 13 000*g* for 5 minutes. Transfer 10 µL to a new slide that has been cleaned with ethanol (EtOH) and add 5 µL of vectashield mounting medium with 2‐(4‐Amidinophenyl)‐6‐indolecarbamidine dihydrochloride, 4′,6‐Diamidino‐2‐phenylindole (DAPI). The slides were carefully cover‐slipped and images were collected at 40× using an Olympus FV1000 laser scanning confocal microscope equipped with Fluoview SV1000 imaging software.

### 2.11 Statistical analysis

Behavioural data, image analyses and microbiome data are presented as mean ± SD Statistical analysis was performed using Statview (Abacus Corporation). The results for the assays in this study were analysed using one‐way ANOVA with Bonferroni post‐hoc analysis. A value of *P* < 0.05 was considered significant.

## 3 RESULTS

### 3.1 Effects of CBP on motor, neurological and gastrointestinal function in PD animals

**Figure 2:**
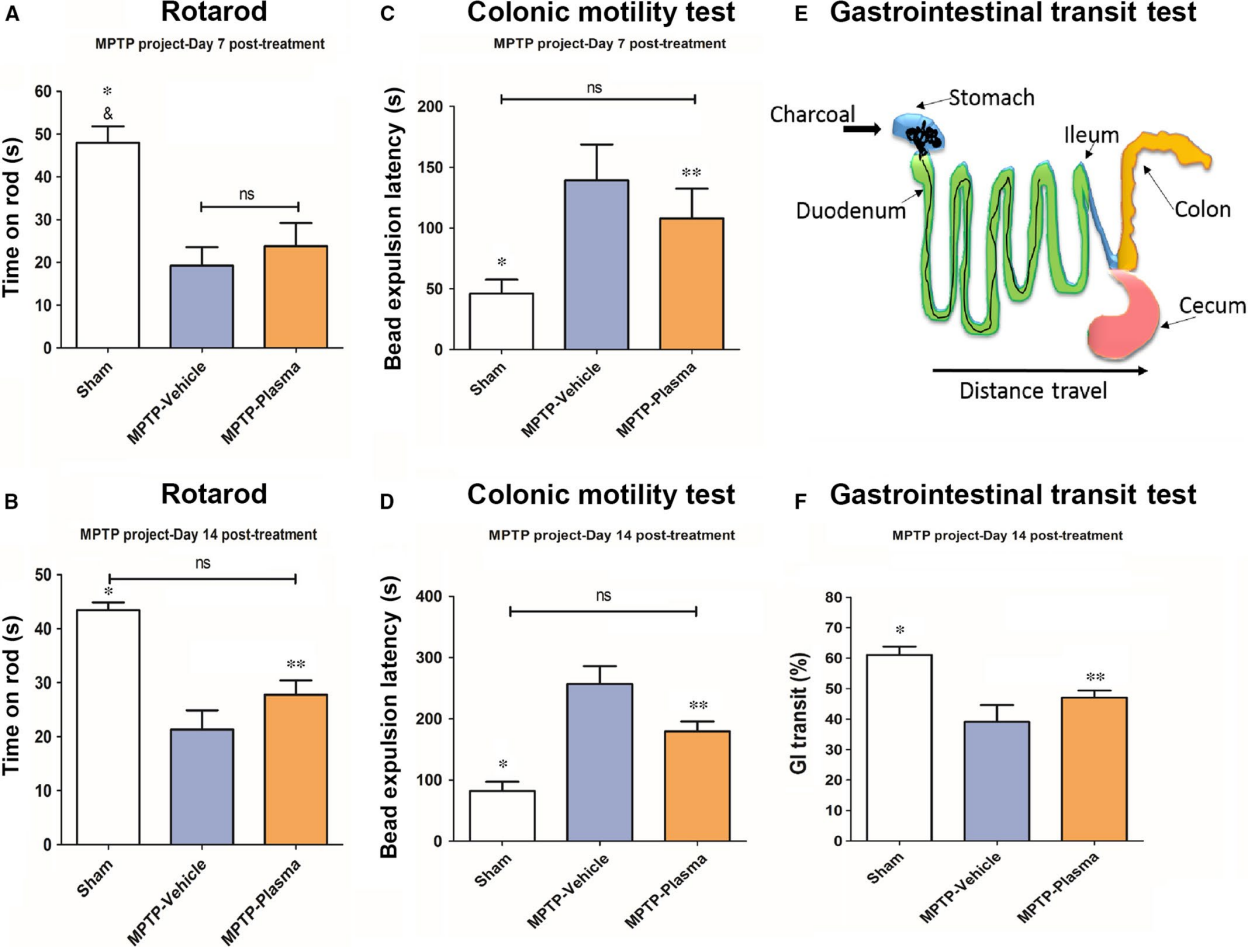
Effect of cord blood plasma on motor, neurological and gastrointestinal function in Parkinson's disease (PD) animals. The motor and non‐motor related Parkinsonian pathologies were observed in a gut‐induced 1‐methyl‐4‐phenyl‐1,2,3,6‐tetrahydropyridine (MPTP) rat model of PD. Initially, rotarod testing did not show much benefit at 7‐d post‐transplant (A), while significant improvements in motor function were observed at the 14‐d time point (B). Additionally, significant improvements non‐motor PD dysfunction were observed in both time points for colonic motility (C,D) and the end‐of‐study charcoal meal gastrointestinal transits test (F) in MPTP lesioned rats that received CBP treatment. *,&: *P* < 0.05, **: *P* < 0.01

The efficacy of multiple injections CBP was investigated in a gut‐induced MPTP rat model of PD. As no adverse reactions were observed in any of the animals receiving iv administration of CBP all animals remained in the study until completion. Results showed that animals which received CBP after the MPTP insult displayed a potential improvement in motor function as revealed by rotarod test compared to the vehicle group at 7 day time point (Figure 2A), with this effect reaching significance (*P* < 0.01) by the 14 day time point (Figure 2B). Additional observations of neurological function consisting of forelimb akinesia did show the generation of PD‐like neurological dysfunction, but did not show any notable improvements by the end of the study (data not shown). Paw grasp testing to determine the extent of motor function deficits in this gut‐MPTP model of PD showed a strong trend towards significance starting around the time of the second CBP administration and continued to the 14 day time point (data not shown).

To determine the efficacy of CBP to reduce the parkinsonian related changes to the gastrointestinal (GI) system, colonic motility and GI transit tests were performed. Colonic motility was observed by inserting a small glass bead into the animal's rectum and measuring the amount of time needed to expel the bead. Testing at day 7 showed a slight decrease in the average time necessary for bead expulsion in animals that received CBP vs vehicle controls (Figure 2C), with significant (*P* < 0.01) reductions observed at both the 7 and 14 day time points (Figure 2D). Additionally, GI transit was observed by providing animals with a charcoal meal, by gavage, approximately 30 minutes prior to euthanasia. During tissue collection the distance the charcoal meal traveled within the intestines was measured (Figure 2E). A significant (*P* < 0.01) restoration in GI transit of the charcoal meal was observed in animals that received CBP vs vehicle controls (Figure 2F).

### 3.2 Effects of CBP on the histopathology of PD brain and gut

**Figure 3:**
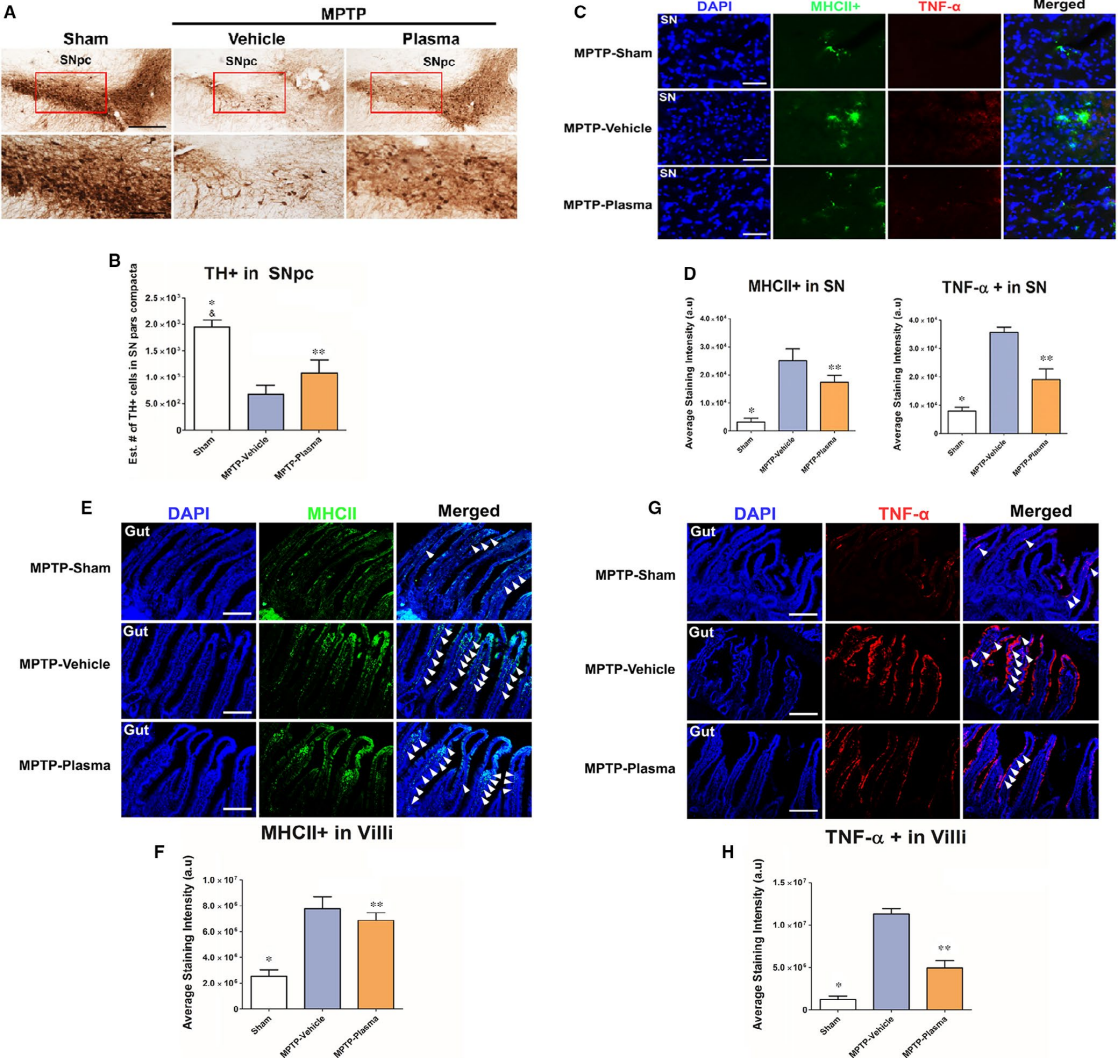
Effect of cord blood plasma (CBP) on the histopathology of Parkinson's in the brain and gut. On day 14 following induction of Parkinson's disease pathology with intraperitoneal injections of 1‐methyl‐4‐phenyl‐1,2,3,6‐tetrahydropyridine (MPTP) the therapeutic effect of CBP treatment was observed in brain and gut tissues. MPTP lesioned rats treated with CBP noted significant reduction in TH‐cell depletion in the substantia nigra (A,B) paired with a significantly decreased inflammatory response as evidenced by decreased major histocompatibility complex (MHCII) and tumour necrosis factor alpha (TNF‐α) intensity in the brain of CBP‐treated animals (C,D). Similarly, the inflammation of the gut found to be significantly reduced through decreased MHCII (E,F) and TNF‐α (G,H) intensity present in the intestinal villi of animals treated with CBP. These findings parallel the trend of behavioural improvements in these animals that received intravascular injections of plasma. *,&: *P* < 0.05, **: *P* < 0.01 Scale bar in A is 50 µm. Scale bar in C, E, G is 100 µm

Histopathological staining of brain tissues was performed to document changes in dopaminergic neuron populations in the SNpc. Additionally, immunofluorescence identifying immune cell activation, and TNF‐α expression in both the brain and gut tissue was performed. Staining for the enzyme TH, a marker of dopaminergic neurons, revealed a reduction in the extent of dopaminergic neuron cell loss in animals given CBP vs vehicle controls (Figure 3A). Image analysis of this TH staining revealed that animals that received CBP vs vehicle controls showed a significant (*P* < 0.01) reduction in dopaminergic depletion (Figure 3B). Additionally, the presence of immune cells populations (MHCII) in the SNpc was significantly (*P* < 0.01) reduced in response to CBP treatment (Figure 3C), with the staining for TNF‐α also showing significant (*P* < 0.01) reductions in the expression of this pro‐inflammatory cytokine in MPTP lesioned animals that received CBP vs vehicle controls (Figure 2D). These effects were mirrored in the gut tissue of these animals with a significant (*P* < 0.01) reduction of both MHCII positive immune cells and TNF‐α cytokine production in animals treated with CBP vs vehicle controls (Figure 3E and 3, respectively).

### 3.3 Effects of CBP on populations of inflammatory microbiota in PD animals

**Figure 4:**
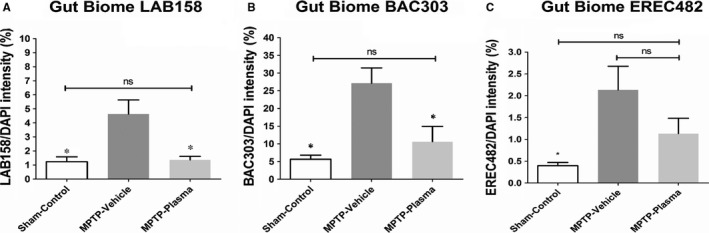
Effect of cord blood plasma (CBP) on populations of inflammatory microbiota. In tandem with the behavioural and histological assays, we also assayed the gut for three biomes associated with inflammation. The levels of (A) LAB158, (B) BAC303 and (C) EREC482 were detected using a fluorescent in‐situ hybridization assay, and found to be elevated at day 14 following 1‐methyl‐4‐phenyl‐1,2,3,6‐tetrahydropyridine (MPTP) lesion in lesioned animals treated with vehicle. These levels were reduced in lesioned animals that received CBP, with significant reductions found for both LAB158 and BAC303, suggesting that the plasma exerted anti‐inflammatory effects in the gut that may have contributed to the observed functional recovery. *,&: *P* < 0.05

The prevalence of several potentially harmful microbiota species were investigated using FISH. While the levels of LAB158, BAC303, and EREC482 were found to be elevated at day 14 following MPTP lesion in lesioned animals treated with vehicle. Significant (*P* < 0.05) reductions in the amount of LAB158 and BAC 303 were observed in the gut of MPTP animals that were given multiple injections of CBP vs vehicle control (Figure 4A, 4). While the population of EREC482 also decreased in response to CBP treatment this observation was not found to carry significance (Figure 4C).

## 4 DISCUSSION

In this study, the potential of CBP as a viable therapeutic agent for both motor and non‐motor symptoms in the gut‐induced MPTP rat model of PD was evaluated. Tests of motor function, neurological behaviour and gastric motility were employed to investigate the therapeutic efficacy of CBP. The ability of CBP to reduce nigral dopaminergic cell loss, while preventing both immune cell activation and pro‐inflammatory cytokine production was also determined. The effect of CBP on the elevated levels of inflammation‐associated microbiota in the gut was examined and compared to levels observed in the vehicle group. The major study findings were that treatment with CBP: (a) significantly improved motor (rotarod) and non‐motor (colonic motility; GI transit) function in PD animal; (b) significantly reduced the amount of dopaminergic (TH) cell loss in the SNpc; (c) significantly reduced activation of immune cells (MHCII) and reduced expression of TNF‐α in both brain and gut tissues; and (d) significantly decreased populations of inflammatory gut microbiota. To our knowledge, this is the first study investigating the potential for CBP to be used therapeutically for the reduction of PD‐related gut pathology.

In the development of treatments for PD, dopaminergic cell replacement has been widely considered as the most straightforward and appealing approach. The first transplantation of fetal derived dopaminergic cells into PD patients resulted in not only survival of the transplant but also integration into the host dopaminergic network.^29^, ^30^ Unfortunately only modest improvements were observed with decreasing positive effects seen over the long‐term in transplanted PD patients.^31^, ^32^ Additionally, a small group of patients that received fetal dopaminergic transplants displayed worsening dyskensias.^33^ While the strategy of conventional dopaminergic cell replacement is still being pursued,^34^ other effects relating simply to the transplantation of stem cells appears to produce therapeutic benefit both peripherally and centrally. These secondary effects, previously examined in animal models of PD,^35^, ^36^ seems to be primarily attributable to alterations in the gut microbiome that reduce inflammation. This in turn may reduce dopaminergic cell loss and promote amelioration of parkinsonian symptoms. The anti‐inflammatory properties of CBP observed in this study produce similar by‐stander effects, which reduce some gut related non‐motor PD deficits. Extending the study over a longer time period with higher doses of CBP may allow for the enhancement of these by‐stander effects which may aid in preventing dopaminergic cell loss and allow for restoration of neurological function.

While CBP shows promise as a potential therapeutic to treat the inflammatory gut observed in PD, the combination of CBP with cord blood stem cells could be the next step in developing a novel PD treatment. Previous studies have already shown that cord blood stem cells are not only effective at extending the lifespan of PD animals,^37^ they also aid in retention of dopaminergic cells within the substantia nigra.^38^ Additionally, CBP has been shown to be an effective supplement in human primary cell cultures.^7^, ^8^, ^9^, ^10^ Substitution of standard cord blood stem cell transplant diluents with CBP might provide a more supportive microenvironment that enhances stem cell survival and increases the therapeutic efficacy of the transplanted cells.

This study offers many novel observations that should lay the foundation for future mechanism and optimization studies of CBP. First, the findings show that cell‐free CBP was effective in attenuating PD symptoms circumvents a major logistical hurdle of generating dopaminergic cells when contemplating large clinical trials of cell transplantation. A basic translational gating item in the use of stem cells for PD is the perceived requirement to generate ample supply of neural stem cells with dopaminergic phenotype. Thus, the present observation that CBP may afford robust functional recovery in PD circumvents the logistic requirement of generating stem cells and differentiating them into dopaminergic cells. Second, the brain region of interest to date for PD studies was for the most part limited to the brain, yet accumulating evidence has implicated a pathological crosstalk between the degeneration of non‐dopaminergic and the dopaminergic system in mediating PD non‐motor symptoms.^39^, ^40^, ^41^, ^42^ Indeed, a paradigm‐shifting hypothesis has been put forward that dopamine segregation in the striatum^43^ and likely the substantia nigra as well, may not fully capture the synaptic plasticity dysfunctions in PD. The propagation of neurodegeneration beyond the nigrostriatal dopamine pathway may suggest a new venue for PD pathology and its treatment. Since the gut microbiome has recently been linked to a number of neurological disorders, our study implicates the gut microbiome as an appealing target for CBP, as well as stem cell fate and cytokine profiling in understanding a non‐dopaminergic and non‐CNS organ contribution to PD pathology. Equally important is the recognition that CBP may contribute to the by‐stander effects of cell therapy in PD by targeting the gut microbiome. Along this line of investigation, such by‐stander effects were previously examined in PD rats^35^, ^42^ and primates.^36^ The extent to which the stem cell‐secreted therapeutic substances reach the striatum and substantia nigra following intravascular transplantation will be of valuable translational and optimization guidance in advancing CBP and stem cell‐based therapy,^36^, ^44^ especially for targeting the gut microbiome in PD treatment.

In conclusion our study results show that multiple administrations of CBP in a MPTP gut‐induced rat model of PD effectively reduced motor and non‐motor dysfunction, which were accompanied by suppression of the pro‐inflammatory cytokine production in both the brain and gut. A decrease in prevalence of several harmful microbiotic species found in the gut of these animals was also observed. While additional motor and neurological testing, such as paw grasp testing and forelimb akinesia, were primarily used in this study to validate the generation of PD‐like motor dysfunction in an intraperitoneally injected MPTP rat model, some trends of amelioration of motor deficits resulting from multiple injections of CBP were observed (data not shown). Interestingly, the therapeutic effects of iv CBP administration appear not limited to the brain function, but attenuate non‐motor symptoms (gut motility) as well. These findings indicate that additional studies investigating both a longer therapeutic time period and potentially larger doses of CBP should be investigated as these modifications may improve therapeutic efficacy. Additionally, that CBP robustly restored the non‐motor gut functions in PD suggests that monitoring of specific physiological indices such as body weight and food consumption may be essential in validating these GI effects. Optimization of CBP timing of treatment, doses and routes of delivery as stand alone or in combination with cord blood stem cells may provide the most effective and safe cell‐based regenerative medicine necessary to sequester the inflammation‐plagued brain degeneration and gut microbiome that plagues PD and various neurodegenerative diseases.

## CONFLICT OF INTEREST

PRS is a co‐founder for Saneron CCEL Therapeutics, Inc. JE is the Director of Research and Development for Saneron CCEL Therapeutics, Inc. PRS and CVB have patents for the application of hUCB as a cell therapy for several disorders.

## AUTHORS CONTRIBUTION

JYL, JPT, JE, PRS and CVB conceptualized, wrote, revised and approved the final version of the manuscript. JYL, JPT and CVB conducted the experiments, collected and analysed the data. JYL, JPT, JE, PRS and CVB interpreted the data.

## Data Availability

All data reported herein are stored in the Department of Neurosurgery and Brain Repair at the University of South Florida Morsani College of Medicine, and will be available upon request with appropriate end‐user agreement.
